# Stability of Porcine Epidemic Diarrhea Virus on Fomite Materials at Different Temperatures

**DOI:** 10.3390/vetsci5010021

**Published:** 2018-02-13

**Authors:** Yonghyan Kim, Venkatramana D. Krishna, Montserrat Torremorell, Sagar M. Goyal, Maxim C.-J. Cheeran

**Affiliations:** Department of Veterinary Population Medicine, College of Veterinary Medicine, University of Minnesota, 225 VMC, 1365 Gortner Ave, St. Paul, MN 55108, USA; kimx4078@umn.edu (Y.K.); vdivanak@umn.edu (V.D.K.); torr0033@umn.edu (M.T.); goyal001@umn.edu (S.M.G.)

**Keywords:** porcine epidemic diarrhea virus, fomite, stability, material, temperature, survival, swine, rubber, plastic, Styrofoam

## Abstract

Indirect transmission of porcine epidemic diarrhea virus (PEDV) ensues when susceptible animals contact PEDV-contaminated fomite materials. Although the survival of PEDV under various pHs and temperatures has been studied, virus stability on different fomite surfaces under varying temperature conditions has not been explored. Hence, we evaluated the survival of PEDV on inanimate objects routinely used on swine farms such as styrofoam, rubber, plastic, coveralls, and other equipment. The titer of infectious PEDV at 4 °C decreased by only 1 to 2 log during the first 5 days, and the virus was recoverable for up to 15 days on Styrofoam, aluminum, Tyvek^®^ coverall, cloth, and plastic. However, viral titers decreased precipitously when stored at room temperature; no virus was detectable after one day on all materials tested. A more sensitive immunoplaque assay was able to detect virus from Styrofoam, metal, and plastic at 20 days post application, representing a 3-log loss of input virus on fomite materials. Recovery of infectious PEDV from Tyvek^®^ coverall and rubber was above detection limit at 20 days. Our findings indicate that the type of fomite material and temperatures impact PEDV stability, which is important in understanding the nuances of indirect transmission and epidemiology of PEDV.

## 1. Introduction

Porcine epidemic diarrhea virus (PEDV) is an Alphacoronavirus in the family *Coronaviridae*. This enveloped RNA virus causes highly contagious viral enteritis in swine [1,2]. The virus was first identified in England in 1971 and then in other European countries [3]. From 1982, PEDV outbreaks were observed in Asian countries including Japan, Korea, Philippines, Vietnam, and China. Until 2009, PEDV strains in Asia were genotypically similar to those in Europe. Since 2010, however, PEDV has caused outbreaks in Asia with severe clinical signs and high mortality in suckling pigs, resulting in heavy economic losses to the pig farmers [3,4,5,6,7]. In May 2013, a PEDV strain, genetically related to a Chinese strain, was introduced in the US, possibly by importation of contaminated feed or contaminated feed tote bags [8,9]. The virus spread rapidly across the country causing high mortality in piglets [10]. Over eight million pigs were killed during this outbreak, leading to an estimated loss of 1.8 billion US dollars [11,12,13,14].

Transmission of PEDV primarily occurs by the fecal-oral route, but indirect transmission can occur when an animal comes in contact with inanimate objects (fomites) contaminated with the feces of PEDV-infected animals. Disease outbreaks primarily occur in early fall and winter, but the rationale for seasonality remains unclear [15]. Previous studies indicate that PEDV is stable at 50 °C and at pH between 7.2 and 10.2 [16,17,18,19]. A recent study found that the virus can survive for up to nine months at the center of manure lagoons [20]. We designed this study to evaluate the survival of PEDV on various fomite surfaces at both room temperature (RT) and 4 °C. Inanimate objects such as rubber boots, gloves, coveralls, and other equipment are routinely used on swine farms and have the potential of being contaminated with manure from PEDV infected animals, thereby helping the indirect mode of virus transmission.

## 2. Materials and Methods

### 2.1. PEDV Propagation and Quantification

A strain of PEDV (PEDV USA/Colorado/2013; GenBank accession number KF272920) was propagated on Vero 76 cells (ATCC CRL-1587, Manassas, VA, USA) using Dulbecco’s modified Eagle medium (DMEM) supplemented with 0.5 μg/mL TPCK-trypsin (Worthington Biochemical Corporation, Lakewood, NJ, USA) and 0.3% tryptose phosphate broth (Sigma, St. Louis, MO, USA). The virus was harvested three days post infection (d.p.i.) using one freeze-thaw cycle. After centrifugation at 500× *g* for 10 min, the supernatant was aliquoted and stored at −80 °C. Virus titration was performed on Vero 76 cells’ monolayers contained in 96-well microtiter plates using serial 5-fold dilutions of samples. Virus titer was calculated by the Spearman-Kärber method [21] and expressed as 50% tissue culture infective dose (TCID_50_)/mL.

### 2.2. Application of Virus on Fomites and Assessment of Stability

Circular pieces (approximately 1 cm^2^) of various fomite materials were cut to fit into individual wells of Costar^®^ 24-well cell culture plates (Corning Inc., Corning, NY, USA). Materials used were Styrofoam, nitrile gloves, cardboard, aluminum foil, Tyvek^®^ coveralls, cloth, metal, rubber, and plastic. Cell culture-grown stock virus was applied on each type of material contained in triplicate wells (200 μL of virus containing 2.1 × 10^6^ TCID_50_/mL). Virus applied on the bottom of 24-well plate without any fomite material served as control. The applied virus was air dried for 2 h in a biosafety cabinet (BSC). Sets of virus-contaminated fomites were stored at either at room temperature (~25 °C) or at 4 °C. At various times thereafter (0, 1, 2, 5, 10, 15, 20, and 30 days post application), the surviving virus was eluted from each material in 1 mL of 3% beef extract −0.05 M glycine buffer placed in 50 mL centrifuge tubes. After thorough mixing for 20 s on a vortexer (Scientific Industries Inc., Bohemia, NY, USA), the eluate was filtered through a 0.22 μM membrane filters (Millipore, Billerica, MA, USA) followed by virus titration on Vero cell monolayers. Virus stability on each fomite material was assessed as three independent replicates for all the time points tested. Separate fomite samples were used at every time point, and eluates were processed independently for quantification of infectious virus and viral RNA. To compare the survival of cell culture-adapted virus with that of virus contained in fecal material, a separate experiment was done. Fecal material obtained from PEDV negative pigs was spiked with 200 μL of cell culture propagated PEDV (2.1 × 10^6^ TCID_50_/mL). After thorough mixing, contaminated fecal matter was applied in 200 μL amounts on fomite materials in triplicate. Virus was eluted and titrated as described above for cell culture derived virus.

### 2.3. Quantitative Real-Time RT-PCR

Stability of viral RNA on fomites was also tested by real-time RT-PCR. Briefly, RNA was extracted from the eluates using MagMAX™ 96 Viral RNA isolation kit (Thermo Fisher Scientific, Waltham, MA, USA). Primer pair was designed to amplify a portion of the PEDV S gene with the following sequences: Forward 1910: ACGTCCCTTTACTTTCAATTCACA and Reverse 2012: TATACTTGGTACACACATCCAGAGTCA. PCR amplification was quantified using a FAM labeled probe 1939: FAM-TGAGTTGATTACTGGCACGCCTAAACCAC-BHQ. The primer and probe were added to AgPath-ID™ One-Step RT-PCR reagents (Thermo Fisher Scientific, Waltham, MA, USA) along with 5 μL of extracted total RNA followed by amplification with 7500 Fast Real-Time PCR System (Thermo Fisher Scientific, Waltham, MA, USA) using the following conditions: reverse transcription at 48 °C for 10 min; denaturation at 95 °C for 10 min; 40 cycles of denaturation at 95 °C for 15 s; and annealing at 60 °C for 45 s.

### 2.4. Immunoplaque Assay

Immunoplaque assays were performed monolayers of Vero cells contained in 24-well tissue culture plates. Duplicate wells were infected with 200 μL of 10-fold serially diluted sample eluate or stock PEDV (positive control). Plates were incubated for 1 h at 37 °C, and the inoculum was removed and replaced with infection medium with or without 1% agarose. At 24 h post infection (without agarose overlay) or 72 h post infection (with agarose overlay), the medium was removed from the wells and the cells were fixed with 4% paraformaldehyde for 20 min at 4 °C. All subsequent washes and incubations were done in PBS, containing 5% normal goat serum and 0.3% triton X-100. After three washes and blocking for an hour in buffer, plates were incubated overnight at 4 °C with one of the following primary antibodies: (i) mouse monoclonal antibody for PEDV spike protein (diluted 1:500; Clone 3F12, Median Diagnostics, Chuncheon, Korea); or (ii) mouse monoclonal antibody for PEDV spike protein (diluted 1:500; Clone S1D12, VMRD, Pullman, WA, USA). Wells were then washed three times with wash buffer and incubated for 1 h at room temperature with alkaline phosphatase conjugated anti-mouse IgG secondary antibody (diluted 1:200; Thermo Fisher Scientific, Waltham, MA, USA). After three washes in buffer, plates were incubated for approximately 20 min with 1-Step™ NBT/BCIP substrate solution (Thermo Fisher Scientific, Waltham, MA, USA). Immunostained cells were observed under a light microscope (Nikon, Tokyo, Japan).

### 2.5. Statistical Analysis

Results are presented as mean ± SEM of the three independent replicates of commonly used fomite materials on a farm. Virus sample eluted from each fomite replicate was processed independently by TCID_50_ assay, immunoplaque assay, and real-time quantitative RT-PCR. An ANOVA was used to determine statistical significance between data sets using GraphPad Prism 7 software (Graphpad Software Inc., San Diego, CA, USA).

## 3. Results

### 3.1. Survival of Infectious Virus on Fomite Materials

**Figure 1:**
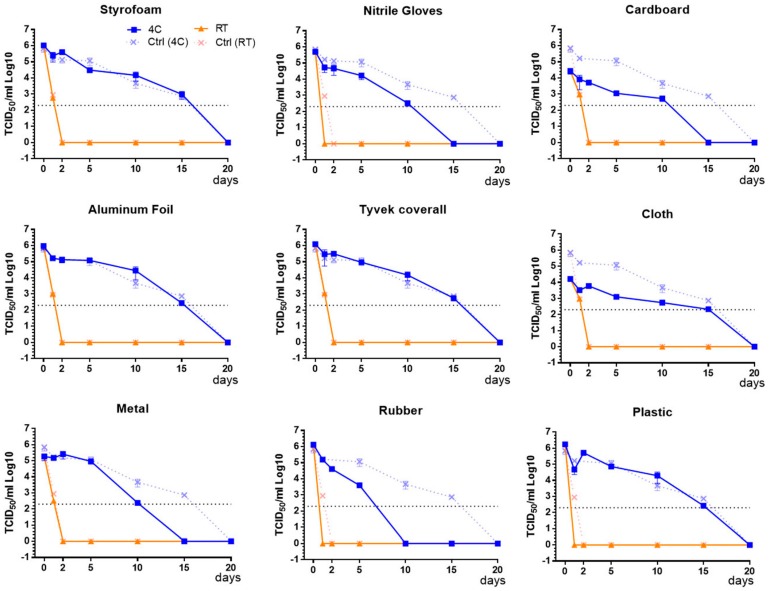
Virus survival on nine fomites at room temperature (RT) and at 4 °C. PEDV was applied on fomite materials or in control wells followed by storage at RT (▲) or at 4 °C (■). The surviving virus was eluted after various time periods and titrated. Decay of infectious virus was rapid at RT but delayed when stored at 4 °C. Black dotted line represents detection limit of TCID_50_ assay, which is 2 × 10^2^ TCID_50_/mL. Data presented are average of three values (± SEM) obtained from independent assessments at each time point indicated.

Infectious PEDV was recovered from fomite materials for up to 15 days post application at 4 °C; only 1 to 2 logs of virus were inactivated during the first 5 days post application (Figure 1). Varying levels of virus loss were observed in different fomite materials used. After 10 days at 4 °C, rubber, nitrile gloves, and metal showed residual virus at or below the detection limit of the assay (≤2 × 10^2^ TCID_50_/mL), while all other materials tested had ≥10^3^ TCID_50_/mL of residual virus. Infectious PEDV was recovered after 15 days at 4 °C on Styrofoam, aluminum foil, Tyvek^®^ coverall, cloth, and plastic, with titers between 10^2^ to 10^3^ TCID_50_/mL. However, virus recovery from nitrile gloves, cardboard, metal, and rubber was below the detection limit of the assay (2 × 10^2^ TCID_50_/mL) after 15 days at 4 °C.

On the other hand, PEDV survival decreased precipitously at room temperature within 1 to 2-days post application, losing 2 to 4 log titers within 24 h (Figure 1).

Infectious PEDV was not recovered from any fomite material after 2 days at RT. Virus recovery from surfaces of Styrofoam, nitrile gloves, aluminum foil, Tyvek^®^ coverall, metal, rubber, plastic, cardboard, and cloth showed no significant differences between the materials at RT, suggesting that storage temperature had a substantial influence on virus survival.

### 3.2. Immunoplaque Assay

**Figure 2:**
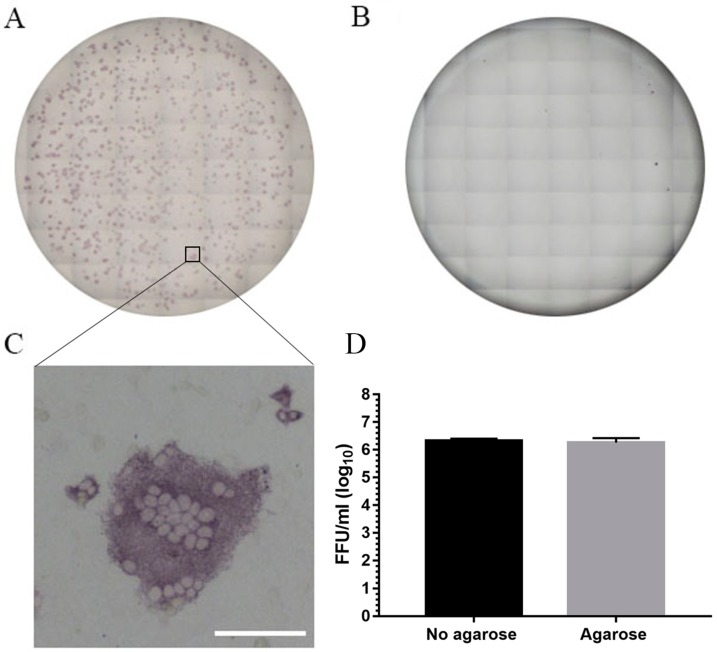
Light microscopic images showing distribution and characteristics of PEDV plaques in immunoplaque assay. (**A**) Virus-positive well showing plaques on Vero 76 cells infected with PEDV; (**B**) un-infected control well; (**C**) high magnification of a typical plaque showing viral-induced syncytium (Scale bar: 100 μm); (**D**) comparison of immunoplaque assay with and without agarose overlay. Data presented are average ± SEM from two independent experiments.

We used a highly sensitive immunodetection method to identify PEDV positive plaques on Vero cells. The lower limit of detection by this method is 24 focus forming units (FFU)/mL. Positive plaques stain purple after immunostaining with an antibody to PEDV spike protein, followed by NBT/BCIP substrate (Figure 2). This assay was performed at 24 h p.i. without using an agarose overlay, because the results obtained with or without agarose were similar.

**Figure 3:**
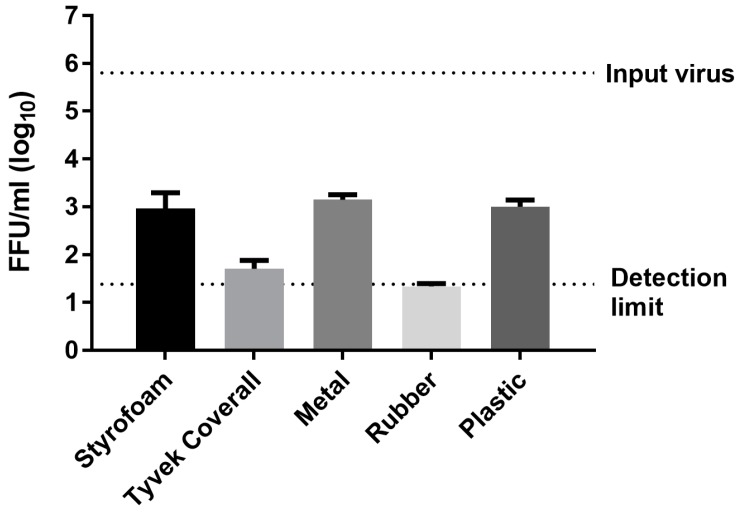
Virus survival on fomite materials at 4 °C. PEDV was applied on fomite materials, stored at 4 °C for 20 days and titered by immunoplaque assay. Input virus titer decreased by 3–5 logs over the course of 20 days. Data presented are average number of FFU ± SEM. The experiment was performed in duplicate using eluates from three independent replicates.

Using the immunoplaque assay, we re-tested virus stability on certain fomite materials after 20 days of storage at 4 °C. Varying levels of viable PEDV were detected (Figure 3). Titers of approximately 1 × 10^3^ FFU/mL were observed in eluates from Styrofoam, metal, and plastic, representing a 3-log virus inactivation after 20 days. The surviving virus on Tyvek^®^ coverall and rubber surfaces was moderately above detection limit (24 FFU/mL). In contrast, no plaques were detected from fomite materials stored at RT for 48 h post application (data not shown).

**Figure 4:**
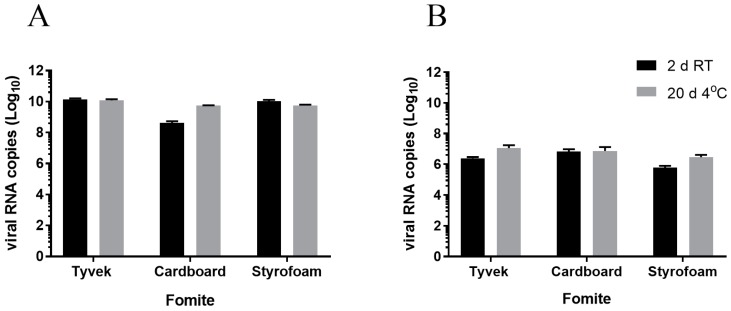
Viral RNA copies on fomite materials. (**A**) PEDV was applied on fomite materials and stored at RT for 2 days or 4 °C for 20 days, and viral RNA copies were determined by qRT-PCR. (**B**) Fecal material was spiked with PEDV, applied on fomite materials and stored at RT for 2 days or 4 °C for 20 days, and viral RNA copies were determined by qRT-PCR. Data representing three fomite materials are presented as average values ± SEM. PCR was performed with RNA obtained from three independent replicates for each material.

To determine the amount of viral RNA remaining on fomites under different storage conditions, eluates were tested by quantitative RT-PCR. Viral RNA was detected after 2 days at RT and 20 days at 4 °C, although no infectious virus was detected at RT after 2 days (Figure 4A). In fact, all materials tested had cycle threshold (Ct) values similar to those of input virus (~16–17), with the exception of eluates from cardboard that showed a Ct value of 21 (Table 1). Quantifiable viral RNA was detected in Styrofoam, Tyvek, and cardboard materials, although infectious PEDV titer decreased by 3 to 4 logs at 4 °C after 20 days. In fecal material spiked with cell culture grown virus, no infectious virus was detected by either TCID_50_ or immunoplaque assay. In fact, eluates from fecal samples induced remarkable cell toxicity, making the infectious virus assays difficult to interpret. However, viral RNA was detected in these samples after 2 days at RT and 20 days at 4 °C; the RNA levels were similar to those of input virus indicating no significant changes in viral genome levels in feces (Figure 4B and Table 1).

## 4. Discussion

Our study demonstrates that cell culture-grown PEDV remains viable for extended periods when dried and maintained in a cold environment. The virus remained viable at 4 °C for up to 20-days on Styrofoam, metal, and plastic, although viral titers decreased by 3 logs in 20 days. When stored at RT, PEDV decreased by 4 to 5 logs within 48 h, rendering it undetectable using infectious virus assays. This observation suggests that the storage temperature of the fomite material has a major impact on virus stability. It appears that low storage temperature delays virus degradation on fomite material. Generally, enveloped viruses are more vulnerable to environmental conditions [22]. However, PEDV was found to have higher stability when stored in spray dried bovine plasma, being stable for up to 3 weeks at 4 °C, 2 weeks at 12 °C, and 1 week at 22 °C [18]. In addition, other coronaviruses, like transmissible gastroenteritis virus (TGEV) and mouse hepatitis virus (MHV), were also found to be more stable at 4 °C, surviving for as long as 28 days at 4 °C and up to 5 days at RT [23]. We demonstrated in this study that the infectious virus decay rate on all fomites increased rapidly at RT. This temperature-sensitive feature of PEDV may be applied to routine procedures in the farms to help eradicate PEDV in the environment and prevent transmission via fomites [16,24,25].

The type of fomite material also has implications on viral stability. For example, the survival of PRRSV (porcine reproductive and respiratory syndrome virus) differed in different types of materials including solid, porous, and liquid substances [26]. We found that infectious PEDV survived at 4 °C on Styrofoam, metal, and plastic, retaining 3 logs of infectious virus (from ~10^6^ FFU/mL) at 20 days. The rate of loss in infectivity was uniform on Styrofoam, aluminum, plastic, and Tyvek^®^ coverall, whereas viral infectivity on metal, nitrile gloves, and rubber dropped rapidly after 5 days at 4 °C. Furthermore, at 0 days post application (i.e., post 2 h of air drying), there was decreased virus recovery from cardboard and cloth, indicating that the composition of the fomite and the porosity of the PEDV-contaminated materials may influence virus survival.

Our results are consistent with previous studies on virus survival on glass, stainless steel, and plastic for up to 10 days [26,27]. Specifically, Styrofoam, metal, and plastic provided extensive PEDV stability. Feed totes made of polypropylene (commonly used in plastic material) allowed PEDV survival for 10 weeks [9]. In addition to storage temperature, porosity of fomite may also influence virus survival and hence virus transmission. Our data suggest that long-term persistence of PEDV on contaminated surfaces could have an epidemiological impact on disease outbreaks, given that >200 infectious virus units were recovered after 20 days at 4 °C on fomite material and that an infectious dose as low as 56 TCID_50_ units infects 100% of animals [28].

PEDV transmission appears to be relatively effective via transportation vehicles [29,30] and feed [18,20,31,32]. Our data showed that PEDV can survive on metal for up to 20 days at 4 °C, which may play a significant role in promoting the spread of PEDV. The longer survival of the virus at cold temperatures may also explain the increase in outbreaks during the winter season [15]. Furthermore, enveloped viruses generally survive better in the presence of organic material [33]. In fact, a large amount of lagoon manure possibly contributes to increased survivability of PEDV, in which the virus can remain infective for up to nine months after being shed by pigs [20].

In this study, viral RNA copy numbers did not correlate with the cell-based assays. It is possible that PEDV infectivity is destroyed by factors that affect the integrity of the viral envelope without affecting RNA degradation at RT. In a recent study, it was shown that PEDV viral RNA steadily degrades over time at temperatures above 37 °C in cell culture medium, but the rapid decline of viral RNA that is detected by PCR occurs only at temperatures higher than 60 °C [34]. Although viral RNA degradation was not assessed in the present study, it is clear that the detection of the viral genome does not affect the infectious nature of the sample.

Besides temperature, the stability of PEDV on fomite is also dependent on presence or absence of other organic material and pH. Although we used both cell culture propagated PEDV and PEDV spiked fecal material on fomites to mimic actual situation in the field, we were unable to determine the survival of the virus in PEDV-spiked fecal material, since the eluates from fomites with feces were not suitable for infectious virus titer due to the cytotoxic effect of the fecal content.

## 5. Conclusions

In conclusion, our findings provide a new perspective on how fomite material and temperature impact viral stability over time, indicating the significance of understanding the nuances of indirect transmission in the epidemiology of PEDV.

## Figures and Tables

**Table 1:** Comparison of immunoplaque assay and qRT-PCR.

Fomite Material	Immunoplaque Assay (FFU/mL)	qRT-PCR (Ct Value) ^†^	
	Cultured		Feces
Input virus	1.0 ± 0.7 × 10^6^	17.06 ± 0.77	29.65 ± 1.79
	Room Temperature/2 days *		
	Cultured		Feces
Styrofoam	<24	16.57 ± 0.34	30.04 ± 0.38
Tyvek coverall	<24	16.65 ± 0.12	28.61 ± 0.49
Cardboard	<24	21.50 ± 0.39	27.56 ± 0.26
	4C/20 days *		
	Cultured		Feces
Styrofoam	9.2 ± 0.6 × 10^2^	17.64 ± 0.51	28.89 ± 0.54
Tyvek coverall	5.0 ± 2.5 × 10^1^	16.93 ± 0.53	27.24 ± 0.75
Cardboard	<24	17.64 ± 0.22	27.69 ± 0.83

Focus forming units (FFU) and cycle threshold values of PEDV in fomite materials, on which cultured PEDV or PEDV-spiked feces were applied and stored at RT or 4 °C. ^†^ cycle threshold (Ct) value ± SD; * day post application.
